# The Minor Allele of rs7574865 in the *STAT4* Gene Is Associated with Increased mRNA and Protein Expression

**DOI:** 10.1371/journal.pone.0142683

**Published:** 2015-11-16

**Authors:** Amalia Lamana, Mercedes López-Santalla, Raquel Castillo-González, Ana María Ortiz, Javier Martín, Rosario García-Vicuña, Isidoro González-Álvaro

**Affiliations:** 1 Servicio de Reumatología, Hospital Universitario de la Princesa, Instituto de Investigación Sanitaria Hospital Universitario La Princesa, Madrid, Spain; 2 Departamento de Inmunología y Oncología, Centro Nacional de Biotecnología, CSIC, Madrid, Spain; 3 Instituto de Parasitología y Biomedicina López-Neyra, CSIC, Granada, Spain; University of Birmingham, UNITED KINGDOM

## Abstract

**Objective:**

The T allele of rs7574865 in *STAT4* confers risk of developing autoimmune disorders. However, its functional significance remains unclear. Here we analyze how rs7574865 affects the transcription of *STAT4* and its protein expression.

**Methods:**

We studied 201 patients (80% female; median age, 54 years; median disease duration, 5.4 months) from PEARL study. Demographic, clinical, laboratory and therapeutic data were collected at each visit. IL-6 serum levels were measured by enzyme immune assay. The rs7574865 was genotyped using TaqMan probes. The expression levels of STAT4 mRNA were determined at 182 visits from 69 patients using quantitative real-time polymerase chain reaction. STAT4 protein was assessed by western blot in 62 samples from 34 patients. To determine the effect of different variables on the expression of *STAT4* mRNA and protein, we performed multivariate longitudinal analyses using generalized linear models.

**Results:**

After adjustment for age, disease activity and glucocorticoid dose as confounders, the presence of at least one copy of the T allele of rs7574865 was significantly associated with higher levels of STAT4 mRNA. Similarly, TT patients showed significantly higher levels of STAT4 protein than GG patients. IL-6 induced STAT4 and STAT5 phosphorylation in peripheral blood lymphocytes. Patients carrying at least one T allele of rs7574865 displayed lower levels of serum IL-6 compared to GG homozygous; by contrast the production of C-reactive protein was similar in both populations.

**Conclusion:**

Our data suggest that the presence of the rs7574865 T allele enhances STAT4 mRNA transcription and protein expression. It may enhance the signaling of molecules depending on the STAT4 pathway.

## Introduction

Signal transducer and activator of transcription 4 (STAT4) is a member of the STAT family, which is activated by the Janus kinase (JAK) family. STAT4 has been described to regulate interleukin (IL) 12, IL-23, and type I interferon (IFN) cytokine signals in T cells and monocytes, leading to T-helper (Th) type 1 and Th17 differentiation, monocyte activation, and production of IFN-ɣ[1]. Consequently, it is conceivable that deregulation of STAT4 activity or expression can alter the functioning of the immune system and, therefore, lead to autoimmune disorders or immunosuppression. In fact, during the last decade, an increasing amount of evidence supports the notion that variants in the *STAT4* gene are associated with an increased risk of developing rheumatoid arthritis (RA), systemic lupus erythematosus (SLE), type I diabetes, Sjögren syndrome, and systemic scleroderma (SSc) [2, 3]. The genetic variant that confers the highest risk of developing autoimmune disorders is expression of the T allele instead of the G allele in the single nucleotide polymorphism (SNP) rs7574865. The functional significance of this change remains unclear, since it occurs in the third intron of the *STAT4* gene, which is a non-coding region. However, since at least five additional SNPs in this intron have also been associated with an increased risk of developing autoimmune diseases, the region must play a relevant role in the regulation of STAT4 biology [2]. Moreover, increased expression of STAT4 mRNA in healthy controls has been associated with the presence of minor alleles of rs7574865, rs3024866, and rs3821236 [4]. In addition, the T allele of rs7574865 has been associated with lower serum levels of IFN-α but a greater IFN-α–induced signature in patients with SLE [5]. A similar finding has been described in hepatitis B virus-related hepatocellular carcinoma from Chinese population [6]. However, increased expression of STAT4 mRNA associated with T allele of rs7574865 has not been reported in patients with autoimmune disorders. Therefore, our objective was to elucidate how the rs7574865 variant of *STAT4* modulates its mRNA expression in a population of patients with early arthritis (EA) and whether it has functional consequences.

## Materials and Methods

### Patients

The study sample comprised 201 patients from the PEARL (Princesa Early Arthritis Register Longitudinal) study. To be eligible, patients had to have had one or more swollen joints for at least four weeks and symptoms for less than a year. Patients with other definite causes of arthritis were excluded. The register protocol included four visits during a two-year follow-up period. At each visit, the data collected and entered into an electronic database were as follows: clinical and demographic data; disease duration at the beginning of follow-up; DAS28 [7]; global disease activity on a 100-mm visual analogue scale scored both by the patient and by the physician; score for the Health Assessment Questionnaire (HAQ) (Spanish version) [8]; and laboratory data, including erythrocyte sedimentation rate (ESR), C-reactive protein (CRP), and rheumatoid factor (RF) determined using nephelometry (positive>20 IU/ml) and anti-citrullinated peptide antibody (ACPA) determined using enzyme immunoassay (Euro-Diagnostica Immunoscan RA; positive >50 IU/ml). In addition, at each visit samples are collected for obtaining DNA, serum and mRNA.

The research carried out in this study is in compliance with Helsinki Declaration. The Ethics Committee for Clinical Research at Hospital Universitario de La Princesa reviewed and approved the protocol, and all patients signed an informed consent form.

### DNA isolation and rs7574865 genotyping

Genomic DNA was isolated from blood samples using the MagNA Pure LC DNA Isolation system (Roche Molecular Biochemicals, Penzberg, Germany). The rs7574865 polymorphism of *STAT4* was genotyped using the TaqMan SNP genotyping assay (Applied Biosystems, Foster City, California, USA; Part number: C__29882391_10). The polymerase chain reaction (PCR) assay was carried out according to the manufacturer’s recommendations. After PCR, the genotype of each sample was attributed automatically by measuring allele-specific fluorescence on a StepOnePlus Real-Time PCR System (Applied Biosystems).

### mRNA isolation and measurement of STAT4 mRNA expression

Total RNA was extracted from peripheral blood mononuclear cells (PBMCs) with the Ultraspect RNA isolation reagent (Biotecx, Houston, Texas). cDNA was obtained with the High Capacity cDNA Reverse Transcription Kit (Applied Biosystems). The expression levels of STAT4 mRNA were determined by quantitative real-time PCR with specific primers in samples from patients (182 visits, 2.64 visits/patient) and from 32 healthy controls (55% female, 58 years median age). Specific mRNA expression was analyzed using SYBR Green PCR Master Mix (Applied Biosystems) in a StepOnePlus Real-Time PCR System (Applied Biosystems). Expression of STAT4 and GAPDH was measured in parallel for each sample, both in patients and in healthy controls. Data from STAT4 mRNA expression were then adjusted for relative gene expression using the 2^-ΔΔCt^ method [9]. The primer sequences were as follows: STAT4 (forward) 5′-TGC AAC CAA AGG AAA TGA AGT-3′, (reverse) 5′-AAT GAA GTT CTT CAG TCA CCA TGT-3′; GAPDH (forward) 5′-GTG AAG GTC GGA GTC AAC G -3′, (reverse) 5′-TGA GGT CAA TGA AGG GGT C -3′.

### STAT4 assessment through western-blot

PBMCs from 62 samples belonging to 36 patients of PEARL study with TT or GG genotype for rs7574865 were lysed at 4°C (30 minutes) in Tris-buffered saline (50 mM Tris-Cl, pH 7.5, 150 mM NaCl) 1% NP40 with a protease inhibitor cocktail (Roche Diagnostics). Whole lysates were analyzed by sodium dodecyl sulfate–polyacrylamide gel electrophoresis (SDS-PAGE), transferred to nitrocellulose membranes, and probed with the anti-STAT4 antibody H.56.9 (Thermo Fisher Scientific) and the anti-*beta-Actin* antibody AC-15 (Sigma-Aldrich) in Tris-buffered saline–Tween 20. Bound antibodies were conjugated with horseradish peroxidase secondary antibodies, and membranes were developed by enhanced chemiluminescence with Super-Signal West Femto chemiluminescent substrate (Pierce Chemical). Densitometric analyses were performed with ImageGauge 3.46 software (Fujifilm).

### IL-6 serum levels measurement

Serum samples are obtained at each visit of PEARL study. Samples are immediately centrifugated and the cell, free supernatant, frozen at –80°C. IL-6 serum levels is routinely measured using the Human IL-6 Quantikine high sensitivity enzyme-immune assay from R&D Systems Europe Ltd. (Abingdon, UK) in samples from those patients with, at least, three visits along the two years follow-up (769 visits; 3.8 visits per patient).

### Assessment of STAT4 and STAT5 phosphorylation induced by cytokines

PBMCs from healthy controls or patients of PEARL study with TT or GG genotype for rs7574865 were stimulated with 10 ng/ml IL-6, 50 ng/ml IL-12 or 100 U/ml IL-2 in RPMI-1640 medium (Gibco BRL) supplemented with 10% fetal bovine serum (Gibco BRL) for 15 minutes at 37°C or unstimulated. The cells were fixed and permeabilized with 1x BD PhosFlow Lyse/Fix Buffer for 15 minutes at 37°C and then with BD PhosFlow Perm Buffer III on ice for 30 minutes. Later, we stained with phycoerythrin (PE) anti-phosphoSTAT4 (pY693), Alexa Fluor 647 anti-phosphoSTAT5 (pY694) or isotype controls (all of BD Biosciences). T cells subpopulation was delineated by staining for CD3 by employing fluorescein isothiocyanate (FITC)-conjugated anti-CD3 monoclonal antibody (BioLegend, San Diego, CA, USA).

### Statistics

Normally distributed variables were described as mean ± standard deviation. The Student’s t test was used to determine whether differences were statistically significant. Non-normally distributed variables were described as median (interquartile range [IQR]), and the Mann-Whitney or Kruskal-Wallis tests were used to compare their distribution between two or more groups, respectively. The correlation between continuous variables was determined using the Pearson test. Qualitative variables were described using an estimation of proportions and compared using the χ^2^ or Fisher exact test.

In order to determine which factors influenced the level of STAT4 mRNA during follow-up, we used the information from the 67 patients whose STAT4 expression levels and full clinical data were available from at least 2 visits. Thus, data from 165 visits (average visits per patient, 2.46) were used to fit a population-averaged model by generalized linear models nested by patient and visit using the *xtgee* command of Stata 12.1 for Windows (StataCorp LP, College Station, Texas, USA). This model allows a better adjustment for independent variables, since by having information from several visits along the follow-up period, it can provide a better estimation of how all the independent variables are associated with the dependent variable. In addition, it is able to consider the number of visits for patient in order to avoid a higher influence of those patients with more visits. Expression of STAT4 was normalized by log transformation using the option *link(log)* of the *xtgee* command (See S1 Fig for information about this transformation). The population-averaged generalized estimating equations were first modeled by including all the variables with a *p* value <0.15 in the bivariate analysis. The final models were constructed using quasi-likelihood estimation based on the independence model information criterion and Wald tests[10], after removing all variables with *p*>0.15.

To assess whether STAT4 protein expression or IL-6 serum level was modulated by rs7574865 genotype we fitted a multivariable analysis using xtgee command of Stata as described above. The variables STAT4 protein expression and IL-6 serum levels were normalized by square root transformation (See S1 Fig for information about this transformation).

## Results

### Patients

At baseline, 143 of the 201 patients (71%) fulfilled the 2010 EULAR/ACR RA classification criteria [11]. The remaining patients were categorized as undifferentiated arthritis (UA). No differences were observed by diagnosis in age at disease onset or gender (median age 54 years, 80% female; Table 1). Patients fulfilling the criteria for RA showed significantly higher baseline disease activity, disability, and CRP and ESR levels. In addition, positive ACPA and RF results were significantly more frequent than in UA patients (Table 1). Median disease duration at study entry was 5.4 months, although it was slightly lower in UA patients (Table 1). No differences in *STAT4* allele distribution were observed between RA and UA patients.

**Table 1:** Baseline characteristics of patients with early arthritis.

	Rheumatoid Arthritis (n = 143)	Undifferentiated arthritis (n = 58)	*p* value
Age (years)	54 (44–70)	53 (39–66)	NS
Female gender (%)	83.2	70.7	0.046
Disease duration (months)	5.4 (3.3–8.4)	4.6 (2.7–8.4)	NS
Smoking ever (%)	40.7	45.3	NS
DAS28-ESR	4.9 (3.8–5.9)	3.7 (2.8–5.1)	<0.001
HAQ	1.125 (0.625–1.625)	1 (0.5–1.375)	0.056
CRP (mg/dl)	0.8 (0.3–1.8)	0.4 (0.2–0.8)	0.012
ESR (mm/h)	30 (20–46)	20 (12–30)	0.002
ACPA-positive (%)	56	19	<0.001
RF positivity (%)	59	15.5	<0.001
STAT4 (%) (GG–GT–TT)	58.2–33.3–8.5	52.6–40.4–7	NS

DAS28-ESR, 28-joint Disease Activity Score; HAQ, Health Assessment Questionnaire; CRP, C-reactive protein; ESR, erythrocyte sedimentation rate; ACPA, anti-citrullinated peptide antibodies; RF, rheumatoid factor; NS, not significant.

The subpopulation used for the study of STAT4 mRNA expression was representative of the whole population (S1 Table).

### Association between the T allele of rs7574865 and higher STAT4 mRNA and protein expression

**Fig 1:**
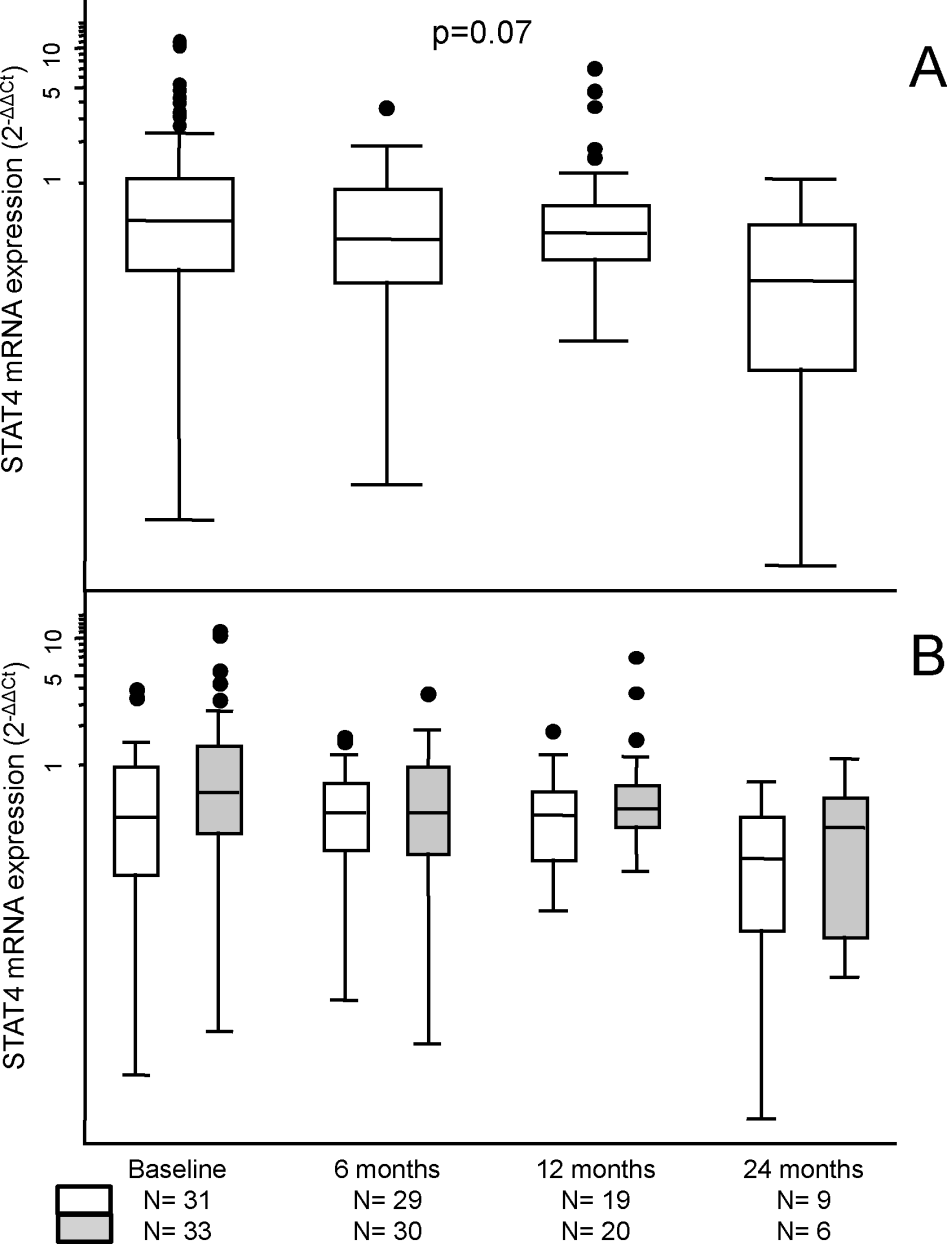
Effect of rs7574865 polymorphism in the STAT4 mRNA levels of patients with early arthritis. (A) Expression levels of STAT4 mRNA during follow-up. Significance level was determined through the Cuzic’s nonparametric test for trend across ordered groups. Statistical significance was considered p<0.05. (B) Data are clustered according to the genotype of rs7574865 in *STAT4*, homozygote GG (white boxes) versus presence of the T allele (gray boxes), and visits during follow-up. Data are presented as the interquartile range (p75 upper edge, p25 lower edge, p50 midline), p95 (line above the box), and p5 (line below the box) of relative STAT4 mRNA expression (2^-ΔΔCt^). Dots represent the outliers.

The expression of STAT4 mRNA in EA patients decreased during follow-up, probably because of an improvement in their disease activity (Fig 1A). This finding was observed both in patients who were homozygous for the common allele (G) of rs7574865 and in those who carried the risk allele (T) of this SNP (Fig 1B). Although a trend toward higher expression of STAT4 mRNA was observed in those patients with at least one T allele, the differences did not reach statistical significance when compared at each visit.

**Fig 2:**
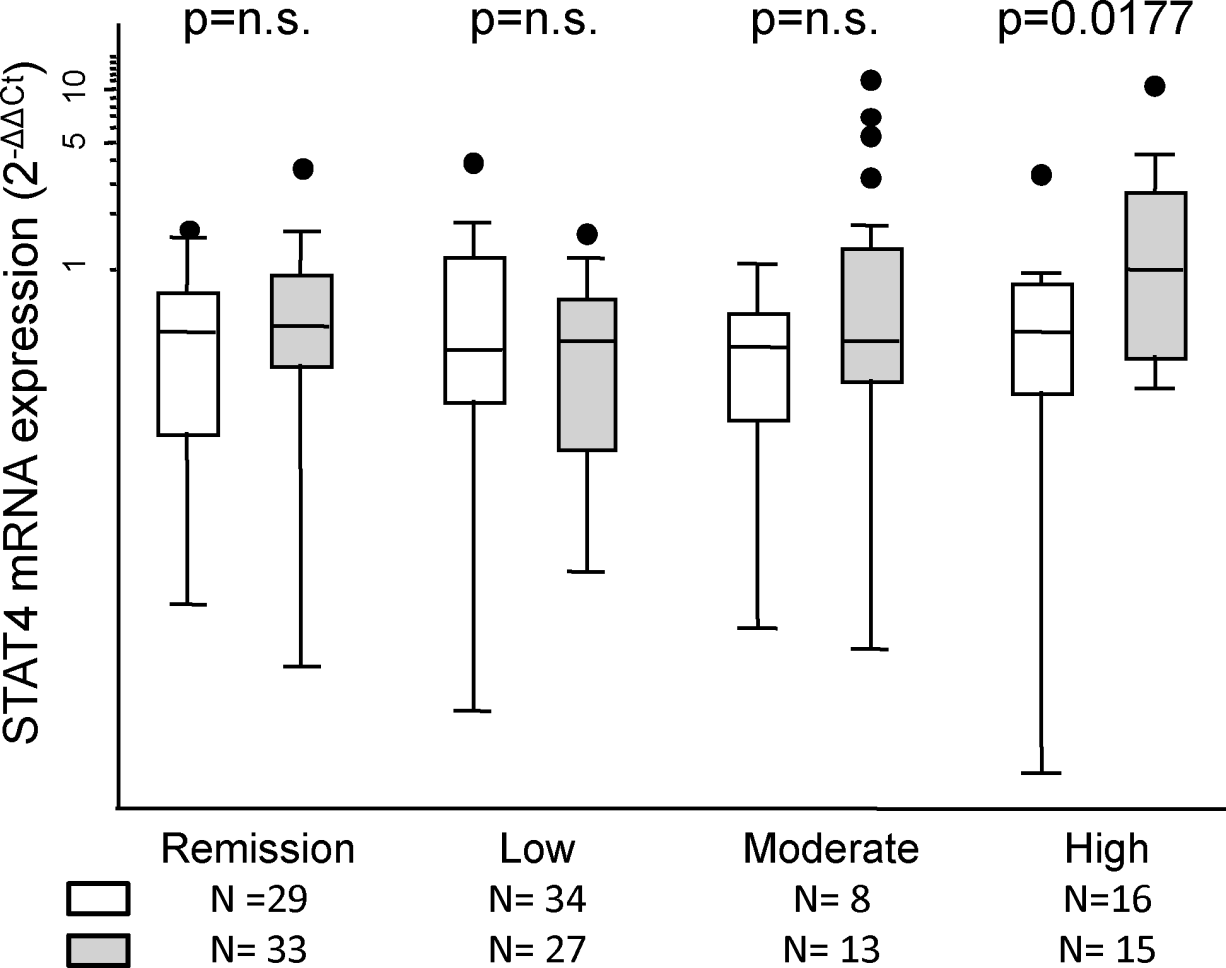
STAT4 mRNA is increased in patients with active early arthritis carrying the T allele of rs7574865. Expression levels of STAT4 mRNA according to the genotype of rs7574865, homozygote GG (white boxes) versus presence of the T allele (gray boxes), and disease activity level based on DAS28. Data are presented as the interquartile range (p75 upper edge, p25 lower edge, p50 midline), p95 (line above the box), and p5 (line below the box) of relative mRNA STAT4 expression (2^-ΔΔCt^). Dots represent the outliers. Significance level was determined through the Mann-Withney test. Due to multiple comparisons, statistical significance was considered p<0.0125 (Bonferroni correction).

Therefore, since the expression of STAT4 could be modified by several confounders, we further analyzed the effect of rs7574865 on the expression of STAT4 mRNA using multivariate analysis (Table 2). Disease activity affected expression of STAT4, with significantly higher levels of mRNA in visits in which patients were at moderate or high levels of disease activity than in visits in which patients were in remission (Fig 2 and Table 2). Furthermore, the use of corticosteroids was significantly associated with lower levels of STAT4 mRNA. In addition, our data revealed that patients older than 65 years expressed significantly higher levels of STAT4 mRNA than those aged less than 45 years. After adjustment for confounders, the data revealed an independent and significant association between the presence of the minor allele of rs7574865 and higher levels of expression of STAT4 mRNA (Table 2 and Fig 2).

**Table 2:** Effect of different characteristics on STAT4 mRNA levels in patients with early arthritis.

		STAT4 Log(2^-ΔΔCt^)	
		β Coeff. (95% CI)	*p* value
Age			
	<45	Ref.	-
	45–65	-0.04 (-0.65–0.58)	NS
	>65	0.90 (0.37–1.43)	0.001
		-	NS
Gender		-	NS
Smoker status			
Diagnosis			
	RA	-	NS
	UA		
ACPA		0.36 (-0.08–0.81)	0.111
RF		-	NS
Disease activity			
	Remission	Ref.	-
	Low	0.17 (-1.07–1.41)	NS
	Moderate	1.28 (0.57–1.99)	<0.001
	High	0.89 (0.10–1.68)	0.023
Treatment (*)			
	Glucocorticoids	-0.14 (-0.26 to -0.03)	0.013
	Methotrexate	-	NS
	Leflunomide	-	NS
	Antimalarial agents	-	NS
	TNF blocker	-	NS
*STAT4* (rs7574865)			
	GG	Ref.	
	GT or TT	1.22 (0.48–1.97)	0.001

Coeff., coefficient; Ref., reference; RA, rheumatoid arthritis; UA, undifferentiated arthritis; ACPA, anti-citrullinated peptide antibodies; NS, not significant.

* The treatment variables correspond to the dose received by the patients at each visit (in mg/day for glucocorticoids, leflunomide, and antimalarial agents; in mg/week for methotrexate; and a dichotomous variable (Yes/No) for TNF blockers).

**Fig 3:**
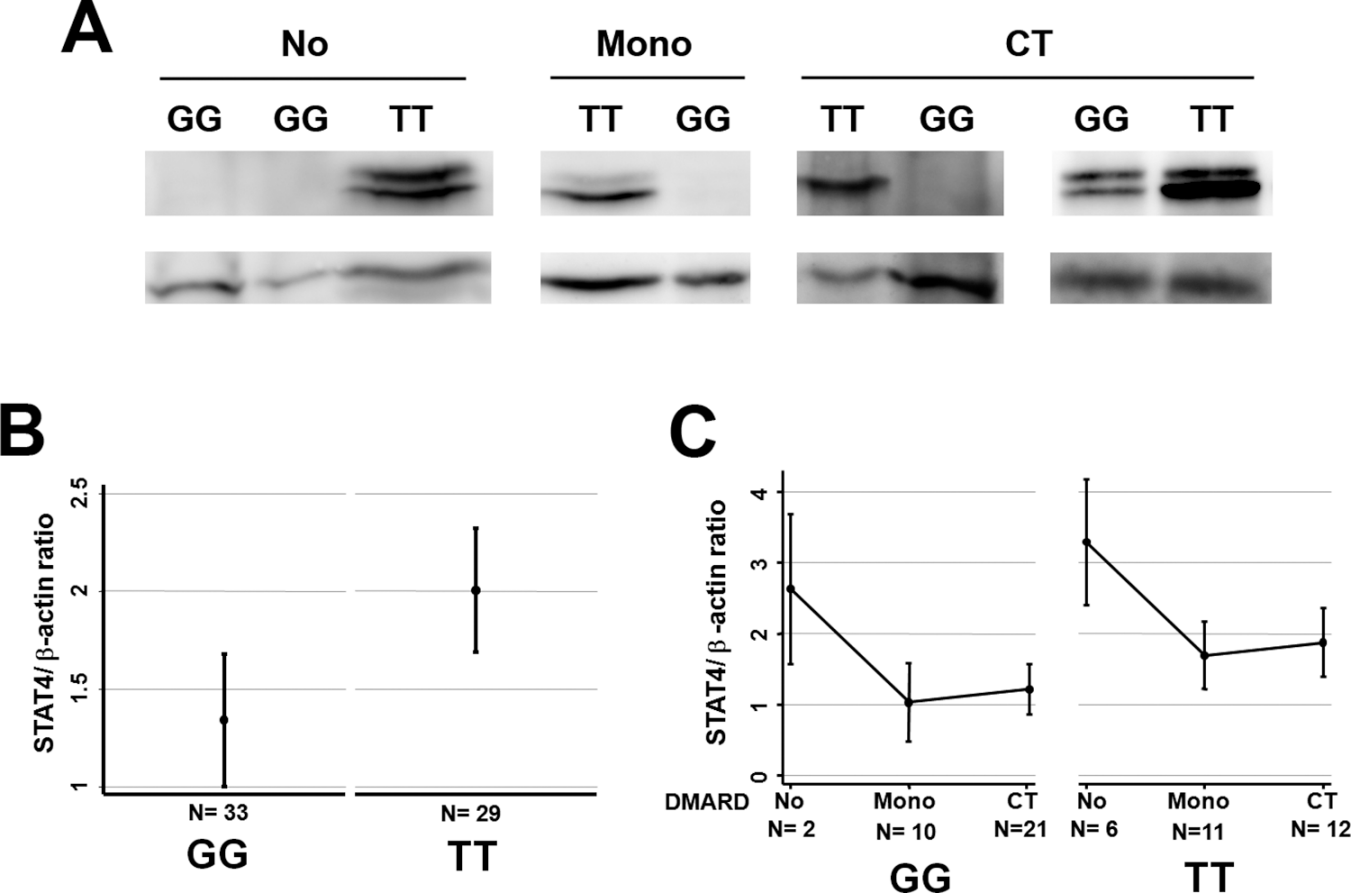
STAT4 protein is increased in patients with early arthritis carrying the TT genotype of rs7574865. A) Western blot analysis of the STAT4 expression in PBMCs from patients with TT or GG rs7574865 genotype with different treatments. Representative blots are shown. B and C) Densitometric quantification of STAT4 protein expression normalized to β-actin expression. The graph represents the linear prediction with 95% confidence intervals of STAT4/β-actin ratio according to the multivariable analysis displayed in Table 3 represented by rs7574865 genotype (B) or by DMARD treatment (No DMARD, Monotherapy or Combined Therapy) and rs7574865 genotype (C).

The protocol of PEARL study does not include storing samples for intracellular proteins analysis. Therefore, we requested permission for using samples obtained to perform routine laboratory controls in patients with rs7574865 TT and GG genotypes in order to measure STAT4 protein expression in PBMCs through western-blot (See S2 Table for information about those patients). Unfortunately, we could not collect information about disease activity at this moment. Those patients in which samples were obtained previous to treatment prescription showed higher STAT4 levels than those treated either with mono or combined therapy of DMARDs (Table 3 and Fig 3C), likely reflecting that disease activity was higher in untreated patients. The use of prednisone was associated with lower levels of STAT4 protein as it happened with mRNA expression (Tables 2 and 3). After adjusting for age, gender, treatment and prednisone dose, patients carrying the TT genotype displayed significantly higher levels of STAT4 expression compared to GG patients (p = 0.009; Table 3 and Fig 3).

**Table 3:** Effect of different variables on STAT4 protein expression in patients with early arthritis.

		STAT4/beta-actin ratio (square-root)	
		β Coeff. (95% CI)	*p* value
Age			
	<45	Ref.	-
	45–65	-0.001 (-0.41–0.41)	NS
	>65	-0.73 (-2.03–0.57)	NS
Gender			
	Male	Ref.	-
	Female	1.61 (0.63–2.59)	0.001
Prednisone (mg/day)		-0.07 (-0.13–0.06)	0.031
DMARD Treatment			
	None	Ref.	-
	Monotherapy	-1.59 (-2.62 –-0.57)	0.002
	Combined therapy	-1.41 (-2.52 –-0.31)	0.012
*STAT4* (rs7574865)			
	GG	Ref.	
	TT	0.66 (0.17–1.16)	0.009

Coeff., coefficient; Ref., reference; NS, not significant.

### Interleukin 6 induces STAT4 phosphorylation

**Fig 4:**
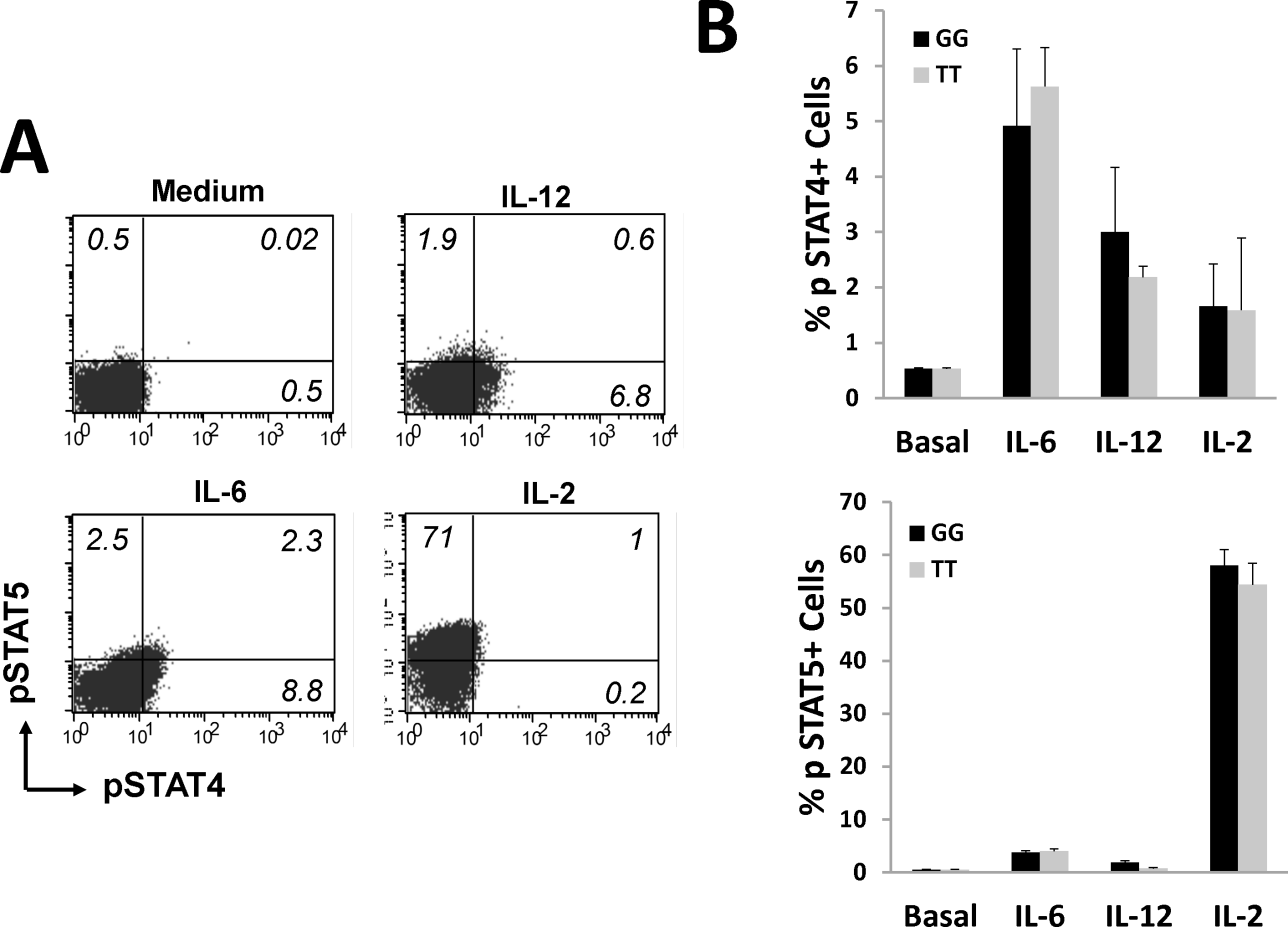
Interleukin-6 induces STAT4 phosphorylation. (A) Representative flow cytometry dot plots showing the expression of phosphoSTAT4 or phosphoSTAT5 positive cells on PBLs unstimulated or stimulated with 10 ng/ml IL-6, 50 ng/ml IL-12 or 100 U/ml IL-2 for 15 min. The percentage of each population is indicated. (B) Percentage of phosphoSTAT4 (upper panel) or phosphoSTAT5 (lower panel) positive PBLs unstimulated (basal) or stimulated with 10 ng/ml IL-6, 50 ng/ml IL-12 or 100 U/ml IL-2 for 15 min. Data are represented as the mean ± sem of 20 patients from PEARL study. Black bars represents patients with GG genotype and grey bar patients with TT genotype for rs7574865; no significant differences were obtained between them (Mann-Whitney test).

Although the signaling of IL-6 is described to be mediated by JAK1 and 2 through phosphorylation of *STAT1*, 3 and 5, considering that JAK/STAT pathway is quite redundant and in PEARL study we routinely measure IL-6 serum levels, we decided to check whether IL-6 induces STAT4 phosphorylation in PBLs. As it is shown in Fig 3, IL-12 stimulation induced mainly a mild STAT4 phosphorylation, probably because a low number of peripheral blood lymphocytes correspond to active Th1 lymphocytes. By contrast, IL-2 caused high *STAT5* phosphorylation and IL-6 was able to induce both STAT4 and 5 phosphorylation (Fig 4). We found no differences between patients with GG or TT genotype for rs7574865 in IL-6 mediated-STAT4 phosphorylation (Fig 4B).

### Patients carrying, at least, one T allele of rs7574865 show similar CRP levels to those homozygous for the G allele despite expressing lower levels of IL-6

**Fig 5:**
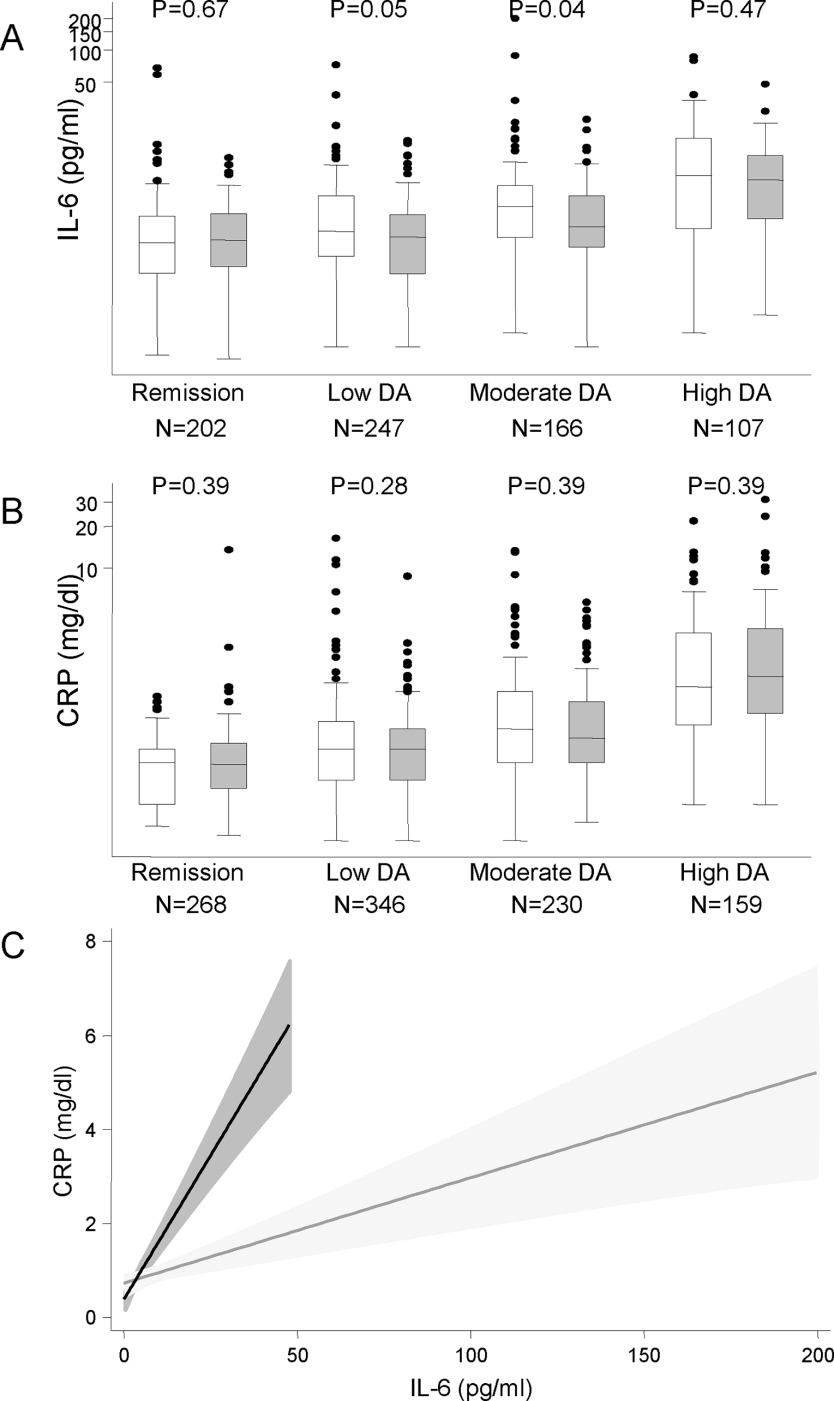
Interleukin-6 (IL-6) induces higher levels of C reactive protein in patients with, at least one T allele of rs7574865. IL-6 (panel A) and C-reactive protein (B panel) serum levels in patients with early arthritis clustered by disease activity level and rs7574865 genotype (white boxes GG genotype, grey boxes GT and TT genotypes). Data are shown as the interquartile range (p75 upper edge of the box, p25 lower edge, p50 midline), as well as the p95 (line above the box) and p5(line below the box). Dots represent outliers. C) Correlation between IL-6 levels and C-reactive protein production depending of rs7574865 genotype. Data are shown as the fitted linear prediction and its 95% confidence interval (clear grey shadow GG genotype, dark grey shadow GT and TT genotypes) using the *twoway* command with the *lfitci* option.

As it has been described that SLE patients carrying the T allele of rs7574865 show increased IFN-ɣ signaling despite lower levels of this cytokine, we comparatively analyzed the levels of IL-6 and CRP depending on rs7574865 genotype. We performed this analysis in view of the data described above suggesting that IL-6 may induce STAT4 phosphorylation. As it is shown in Fig 5A, IL-6 levels increased as it did the disease activity level (p<0.001 for either GG or GT/TT populations; Cuzic’s nonparametric test for trend across ordered groups). When patients were not in remission, those carrying the T allele of this SNP tended to show lower levels of this cytokine. When we analyzed raw data, differences were only statistically significant at low and moderate disease activity levels (Fig 5A). However, adjusting for confounders (disease activity, age, diagnosis, …), we found that carrying the minor allele of rs7574865 was clearly and significantly associated with lower levels of IL6 (Table 4).

**Table 4:** Effect of different variables on IL-6 serum levels in patients with early arthritis.

		IL-6 (square-root [pg/ml])	
		β Coeff. (95% CI)	*p* value
Age			
	<45	Ref.	-
	45–65	0.11 (-0.20–0.42)	NS
	>65	0.47 (0.13–0.81)	0.007
Gender		-	NS
Smoker status			
	Never	Ref.	-
	Ex-	0.41 (0.07–0.75)	0.019
	Current	0.20 (-0.11–0.52)	NS
Disease activity			
	Remission	Ref.	-
	Low DA	0.44 (0.25–0.62)	<0.001
	Moderate DA	0.80 (0.58–1.01)	<0.001
	High DA	1.36 (1.11–1.61)	<0.001
Diagnosis			
	RA	Ref.	-
	UA	-0.43 (-0.72 –-0.14)	0.004
Rheumatoid factor +		-	NS
ACPA +		0.27 (-0.01–0.55)	0.060
Methotrexate treatment			
	No	Ref.	-
	Yes	-0.46 (-0.63 –-0.30)	<0.001
*STAT4* (rs7574865)			
	GG	Ref.	-
	TT	-0.32 (-0.58 –-0.07)	0.012

Coeff., coefficient; Ref., reference; NS, not significant; DA, disease activity; RA, rheumatoid arthritis; UA, undifferentiated arthritis. ACPA, anti-citrullinated peptide antibodies

By contrast, there were no differences by rs7574865 genotype in the level of CRP clustered by level of disease activity (Fig 5B). Interestingly, the pooled fitted linear prediction clustered by rs7574865 genotype showed a clearly higher production of CRP with lower levels of IL-6 in those patients with, at least, one T allele compared with those homozygous for the major allele G (Fig 5C).

## Discussion

To our knowledge, this is the first study to detect increased STAT4 mRNA and protein expressions in patients with an autoimmune disorder carrying the T allele of rs7574865. Our data are consistent with the observation by Abelson et al. that this genetic variant may be associated with increased STAT4 mRNA expression in healthy controls [4]. The relevance of this finding could explain how the minor allele of rs7574865 confers a high risk of developing autoimmune disorders, since it fits well with the previous observation of increased IFN-α-induced signature in SLE patients harboring this genetic variant, despite lower levels of IFN-α in serum [5]. Our data in patients with EA seem to support a similar behavior for IL-6, a cytokine that had not been previously described to induce STAT4 phosphorylation. Other authors considering that STAT4 is involved in the signaling of IL-12, IL-23, and IFN-ɣ, have suggested that patients carrying the rs7574865 minor allele might display stronger Th1 and Th17 cytokine responses [2]. In this regard, we recently reported that patients with EA who are homozygous for the T allele of rs7574865 have a poorer clinical course [12]. Here, we report that patients carrying the minor allele of rs7574865 display similar levels of CRP despite expressing lower levels of IL-6. However, the fact that hepatocytes (and not T cells) are the main source of CRP does not allow us to draw conclusions between the levels of CRP and the relative potency of the Th1 or Th17 response.

The precise mechanism leading to higher expression of STAT4 remains unclear, since rs7574865 is located at the third intron of the *STAT4* gene instead of in the promoter region. Little is known about how expression of STAT4 is regulated at transcriptional level. Published data on STAT4 transcriptional regulation focus on the promoter region 5′ [13], and no data are available on the potential role of introns in the regulation of *STAT4*. Nevertheless, sufficient data indicate that expression is more effective in genes with introns than in intronless genes, since introns have important regulatory functions in gene expression, either through silencing of expression in some tissues or even by increasing expression more efficiently than the promoter [14, 15].

It is difficult to determine the association between genetic variants and their functional consequences when studying complex disorders such as autoimmune diseases. Consequently, the presence of many possible confounders (eg: gender, age, disease activity, and treatment) necessitates complex multivariate analysis. In this regard, our work confirmed the association between disease activity and the intensity of STAT4 mRNA expression, a finding that had previously been suggested by Lü et al [16]. In addition, the use of glucocorticoids was associated with a decrease in levels of STAT4 mRNA and protein expression, and our statistical analysis also suggested that age was associated with increased expression of STAT4 mRNA. This later finding was not confirmed at the protein level. By contrast, female gender appeared to be associated with higher STAT4 protein expression. Therefore, these findings require confirmation in other populations and they should be taken into consideration in the study of these complex disorders.

One of the limitations of our study is the small sample size, as the prevalence of homozygous TT patients is low in our EA population, thus preventing us from establishing whether the effect of the T allele was dose-dependent. However, this issue is offset by the availability of several samples per patient at different follow-up times and the different degrees of disease activity, which allow us to accurately identify the relationship between disease activity and STAT4 mRNA and protein expression. Another possible limitation is that the PCR primers we used detect both α- and β-isoforms of STAT4 mRNA. Nevertheless, the expression of β-transcript is much lower than that of the α-isoform, and both follow the same pattern of expression [4]; therefore, no relevant bias seems to be involved.

## Supporting Information

S1 Fig Histograms obtained by transformation using the command gladder which displays histograms of transforms of the variable (identity) according to the ladder of powers in order to obtain a normally distributed variable. Skewness and kurtosis tests were applied to determine normality. Logarithmic transformation was chosen for STAT4 mRNA expression. Square root transformation was chosen for WB analysis because with log transformed data the analysis did not converged. Square root transformation was the best option for IL-6 serum levels.
(TIF)

S1 Table This table describes the characteristics of the subpopulation used for the study of STAT4 mRNA expression showing that it is representative of the whole PEARL study. (DOCX)

S2 Table This table describes the characteristics of the subpopulation used for the study of STAT4 protein expression showing. In this case only subjects with rs7574865 TT or GG genotype were studied among the patients of PEARL study.
(DOCX)
