# Supplementary figures and images for: The Minor Allele of rs7574865 in the STAT4 Gene Is Associated with Increased mRNA and Protein Expression
Source: PLoS One. 2015 Nov 16;10(11):e0142683. doi: 10.1371/journal.pone.0142683 (PMC4646635; doi:10.1371/journal.pone.0142683)

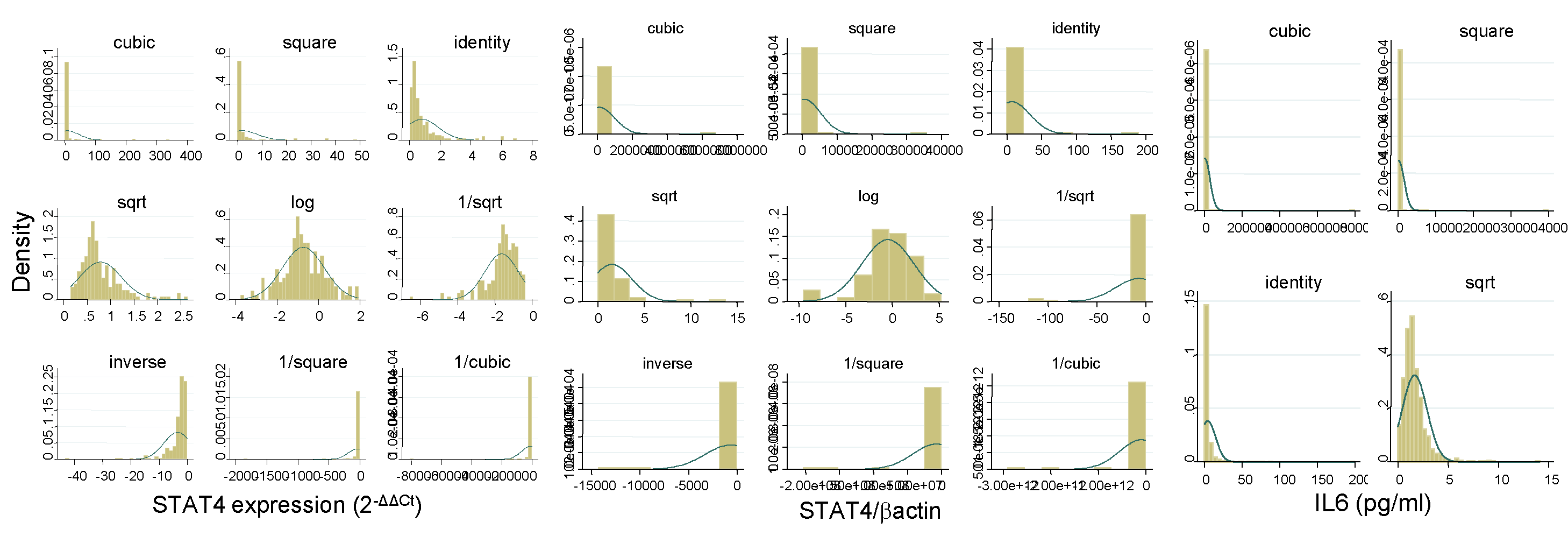

Supplement: S1 Fig — Skewness and kurtosis tests were applied to determine normality. Logarithmic transformation was chosen for STAT4 mRNA expression. Square root transformation was chosen for WB analysis because with log transformed data the analysis did not converged. Square root transformation was the best option for IL-6 serum levels. (TIF) [file pone.0142683.s001.tif]
