# Supplementary material for: The Minor Allele of rs7574865 in the STAT4 Gene Is Associated with Increased mRNA and Protein Expression
Source: PLoS One. 2015 Nov 16;10(11):e0142683. doi: 10.1371/journal.pone.0142683 (PMC4646635; doi:10.1371/journal.pone.0142683)
Supplement: S1 Table — (DOCX) [file pone.0142683.s002.docx]

**Supplementary Table 1.** Baseline characteristics of patients with early arthritis included in the STAT4 mRNA expression study.

|  | Rheumatoid arthritis (n=44) | Undifferentiated arthritis (n=25) | *p* value |
| --- | --- | --- | --- |
| Age (years) | 52 (41 – 69) | 53 (46 – 68) | NS |
| Female gender (%) | 88.6 | 92 | NS |
| Disease duration  (months) | 5.4 (2.8 – 8.4) | 3.9 (2.3 – 6.9) | 0.083 |
| Smoking (%) | 37.5 | 60.9 | 0.073 |
| DAS28-ESR | 5.1 (3.5 – 6.1) | 3.9 (3.0 – 4.8) | 0.01 |
| HAQ | 1.125 (0.625 – 1.875) | 0.875 (0.375 – 1) | 0.044 |
| CRP (mg/dl) | 1.2 (0.4 – 2.7) | 0.3 (0.1 – 0.7) | 0.009 |
| ESR (mm/h) | 35.5 (19.5 – 57) | 21 (12 – 32) | 0.025 |
| ACPA-positive (%) | 68.2 | 12 | <0.001 |
| RF positivity (%) | 75 | 20 | <0.001 |
| *STAT4* (%)  (GG – GT – TT) | 47.6 – 42.9 – 9.5 | 56 – 40 – 4 | NS |

DAS28-ESR, 28-joint Disease Activity Score; HAQ, Health Assessment Questionnaire; CRP, C-reactive protein; ESR, erythrocyte sedimentation rate; ACPA, anti-citrullinated peptide antibodies; RF, rheumatoid factor; NS, not significant.
