# Supplementary material for: The Minor Allele of rs7574865 in the STAT4 Gene Is Associated with Increased mRNA and Protein Expression
Source: PLoS One. 2015 Nov 16;10(11):e0142683. doi: 10.1371/journal.pone.0142683 (PMC4646635; doi:10.1371/journal.pone.0142683)
Supplement: S2 Table — In this case only subjects with rs7574865 TT or GG genotype were studied among the patients of PEARL study. (DOCX) [file pone.0142683.s003.docx]

**Supplementary Table 2.** Baseline characteristics of patients with early arthritis included in the STAT4 protein expression study.

|  | Rheumatoid arthritis (n=26) | Undifferentiated arthritis (n=8) | *p* value |
| --- | --- | --- | --- |
| Age (years) | 50 (38 – 62) | 57 (51 – 65) | NS |
| Female gender (%) | 88.6 | 75 | NS |
| Smoking (%) | 19.2 | 12.5 | NS |
| ACPA-positive (%) | 65.4 | 37.5 | 0.124 |
| RF positivity (%) | 65.4 | 37.5 | 0.124 |
| *STAT4* (%)  (GG –TT) | 61.5 – 38.5 | 62.5 – 37.5 | NS |

ACPA, anti-citrullinated peptide antibodies; RF, rheumatoid factor; NS, not significant.
